# Supplementary material for: Spatial single-cell atlas reveals regional variations in healthy and diseased human lung
Source: Nat Commun. 2025 Nov 5;16:9745. doi: 10.1038/s41467-025-65704-0 (PMC12589588; doi:10.1038/s41467-025-65704-0)
Supplement: Supplementary file 1 — Supplementary Information [file 41467_2025_65704_MOESM1_ESM.pdf]

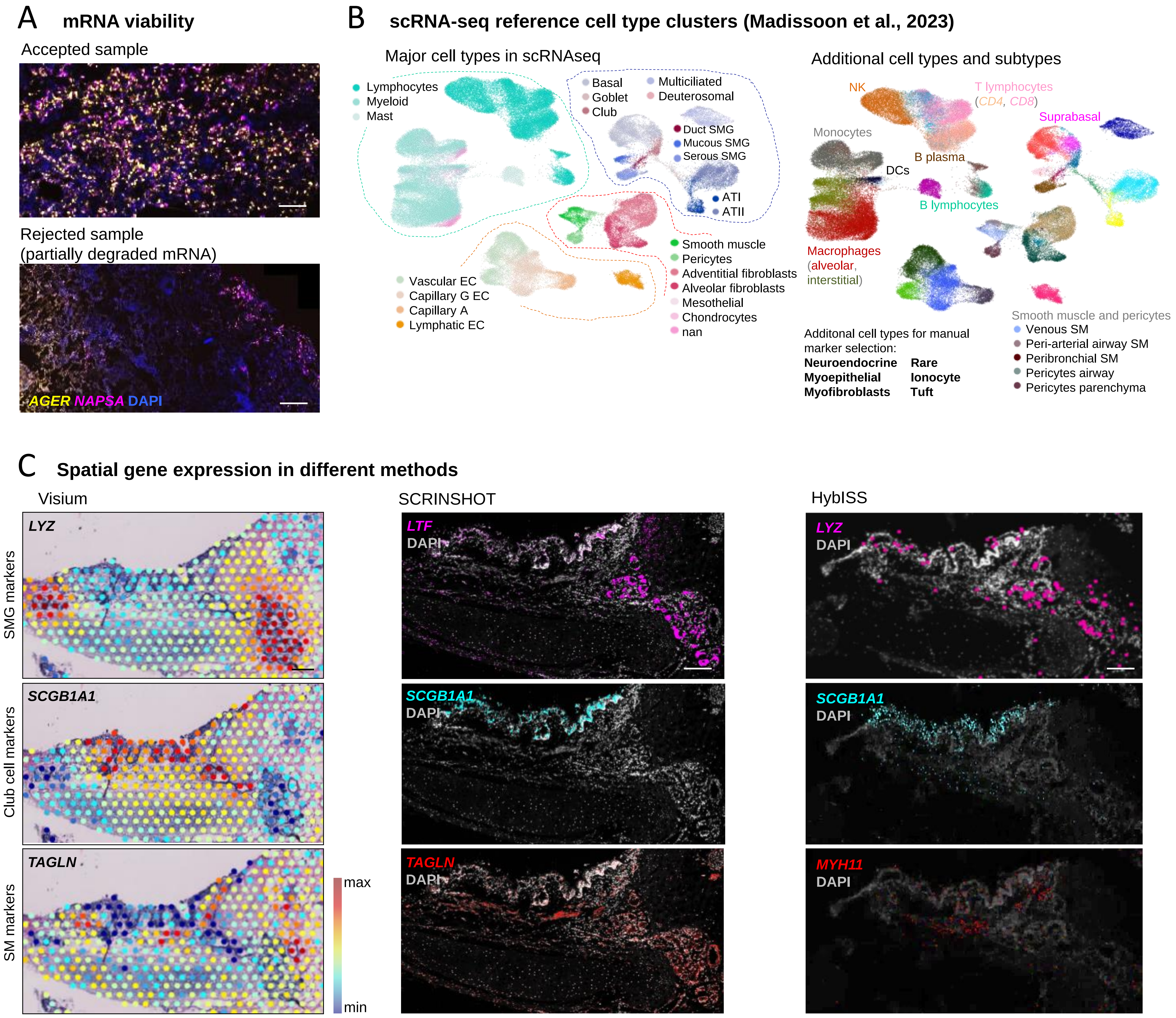

Supplementary Figure 1. Quality control prior to cell type annotations.

A. Representative accepted and rejected samples demonstrating mRNA signal for cell type marker genes for alveolar type I (*AGER*, yellow) and II (*NAPSA*, magenta) cells on top of nuclei (DAPI, blue) in alveolar region tissue sections. Scale bar: 200 µm.

B. Clusters from scRNA-seq datasets [1] used for probe panel selection for targeted methods. Left – cell types targeted with SCRINSHOT marker panel, right – additional cell types targeted with HybISS panel. Additional cell types (not represented in scRNA-seq) based on other publications were targeted based on manual marker selection. SMG – submucosal gland, SM – smooth muscle, EC – endothelial cells, G – general, A – aerocytes, DC – dentritic cells, ATI/II – alveolar epithelial type I or II, NK – natural killer cells.

C. Signal of characteristic markers of the indicated cell types plotted on the images of proximal lung consecutive sections, analyzed with Visium, SCRINSHOT and HybISS. Detected transcripts are shown on top of hematoxylin and eosin staining (Visium) or DAPI (gray, SCRINSHOT and HybISS). Scale bar: 200 µm.

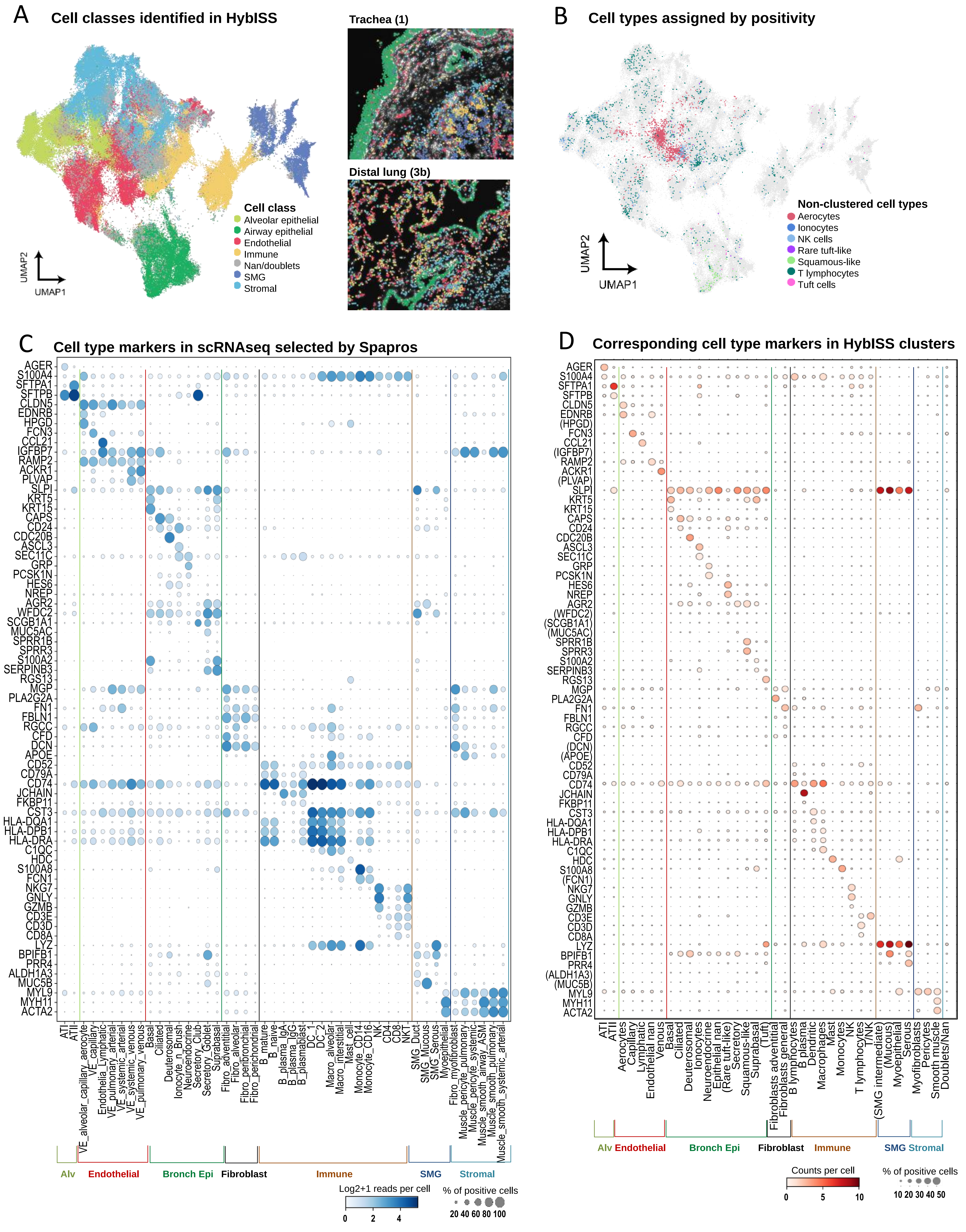

Supplementary Figure 2. Characterization of cell types identified in HybISS analysis.

A. (Left) UMAP plot of cells profiled with HybISS, colored by assigned cell class. (Right) Complementary maps of cell classes identified by HybISS in the trachea and distal lung sections. Colors as in the UMAP plot. Nuclei: gray. Scale bar: 200 µm.

B. UMAP plot of the cells analyzed by HybISS, highlighting the indicated, additional cell types that were manually annotated using distinct marker positivity.

C. Dotplot of the expression of 2-3 top predicted cell type panel markers in the reference scRNA-seq dataset [1]. Color intensity: $\log_2$(normalized UMI-counts+1) (library size was normalized to 10.000). Dot size: percentage of positive cells.

D. Dotplot of the same markers as in (C) in the detected cell types of the HybISS dataset. Color intensity: mean counts per cell. Dot size: percentage of positive cells. Markers with low or sparsely detected expression, and the cell types with low confidence detection, are in brackets. Source data are provided as a Source Data files.

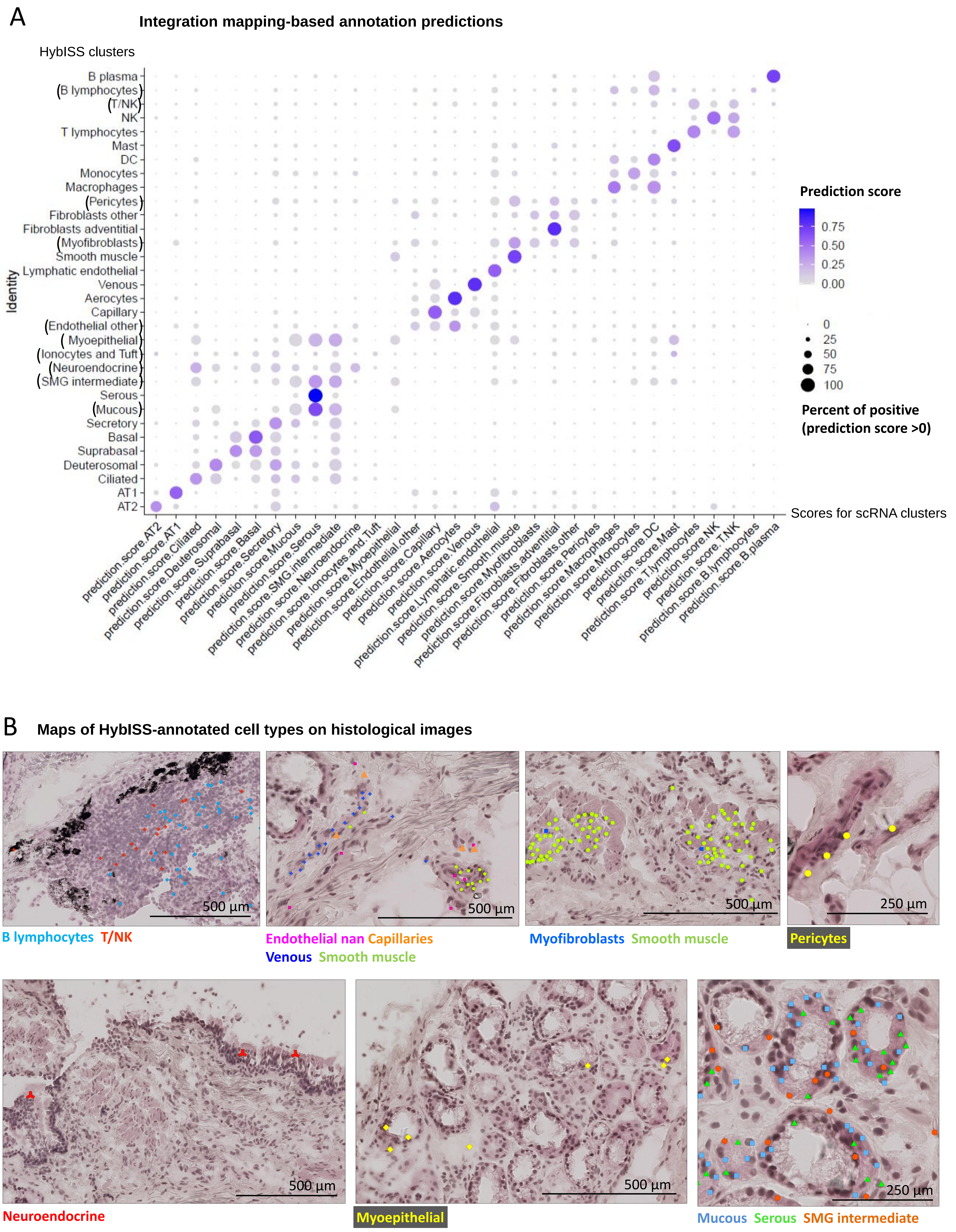

Supplementary Figure 3. Cell type annotation prediction using mapping integration method.

A. Balloon plot of HybISS cell types (Y axis) and their predicted labels from scRNA-seq (X axis, level 2 classification from Madissoon et al). Prediction scores calculated using integration mapping method in Seurat (Hao et al., 2022). Cell types with multiple or unclear predictions are labeled with brackets.

B. Cell types in brackets from (A) mapped on histological tissue images of proximal lung sections. B lymphocytes and T/NK cells are mapped to the immune cell accumulation. Endothelial nan cells appear both in arterial endothelial lining and sparsely in the tissue among capillaries. Myofibroblasts are rare and located in proximity to smooth muscle, potentially being smooth muscle/fibroblast doublets. Pericytes are usually located in proximity to blood vessels, neuroendocrine cells - in the airway epithelium, myoepithelial cells – around submucosal gland. Mucous cells can be observed in the mucous tubules (transparent cytoplasm) of submucosal gland, but occasionally appear in serous tubules (stained cytoplasm), indicating that mucous annotation partially contains serous cells. SMG intermediate is mapped to both serous and mucous tubules of the submucosal gland, and indicates *PRR4*-negative population of serous and duct cells, but not exclusively SMG duct population. Source data are provided as a Source Data file (Integration analysis).

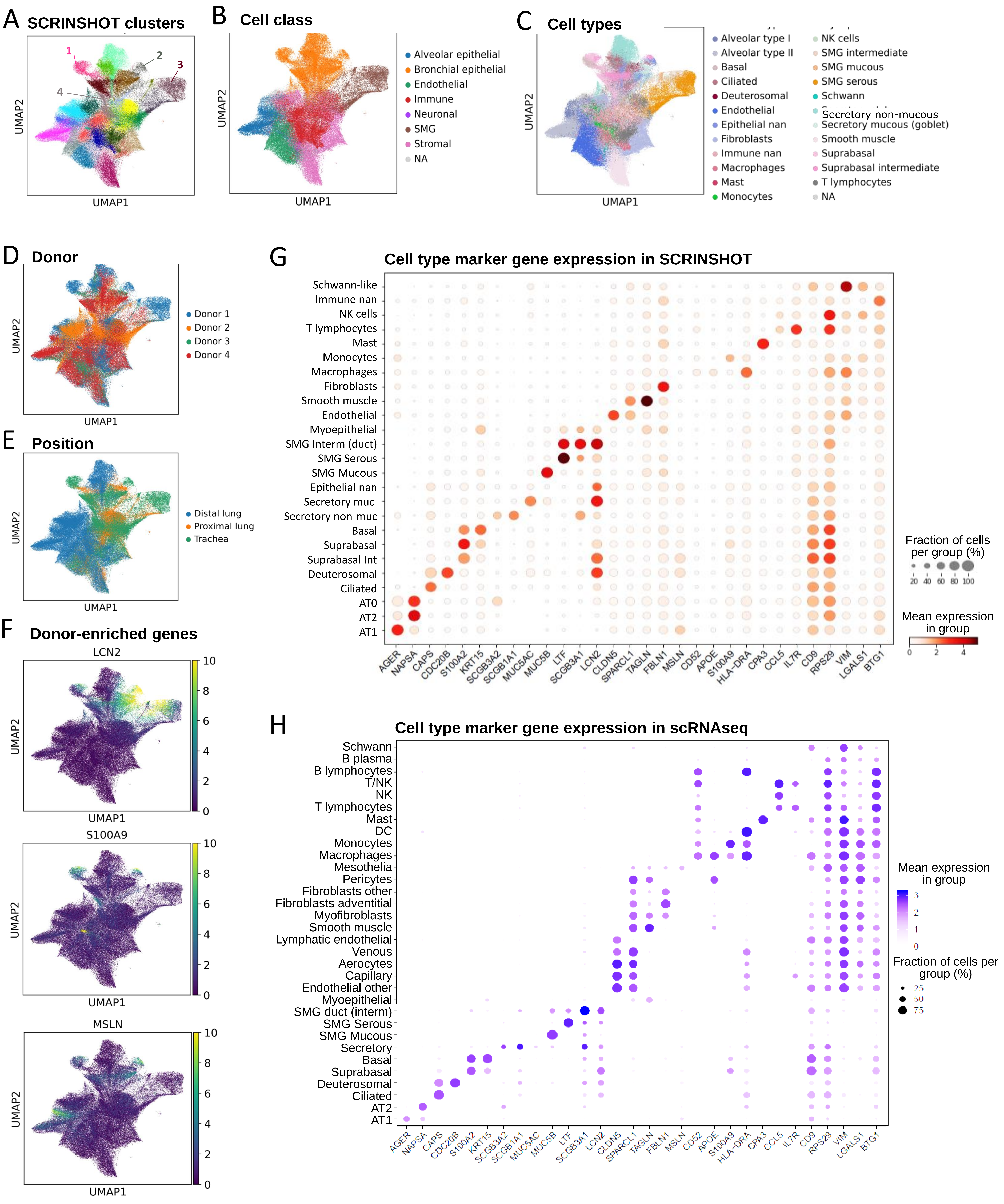

Supplementary Figure 4. SCRINSHOT data clustering and cell type annotation.
A. Clusters of combined data from four donors and three anatomical locations (positions). Four sample-specific epithelial clusters are labeled with numbers.
B-C. Cell class (B) and cell type (C) annotations from single-sample clustering, combined in UMAP from A.
D. Donor numbers metadata plotted on UMAP in A.
E. Anatomical location (position) metadata plotted on UMAP in A.
F. Gene expression of donor-enriched genes LCN2, S100A9 and MSLN, that affected clustering (A, labeled clusters 1-4), despite the presence of different cell types (C) in these clusters.
G. Balloon plot of the expression of marker genes in annotated SCRINSHOT-based cell types (using single sample clustering). Data from four donors.
H. Expression of the marker genes from (G) in scRNA-seq data from Madissoon et al [1] with level 2 annotations. Data from eight donors.

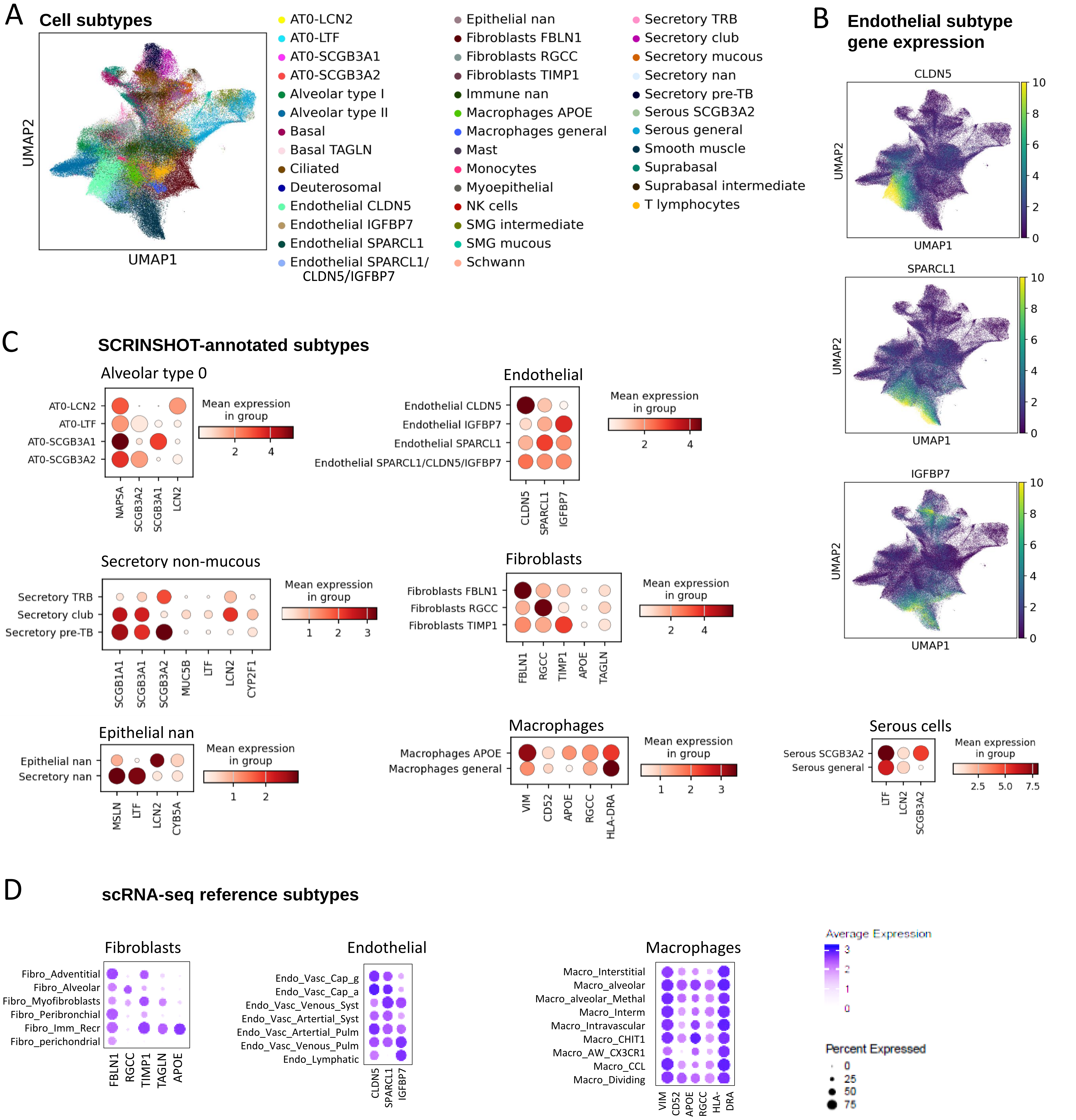

Supplementary Figure 5. Cell subtype annotation in SCRINSHOT data.

A. Cell subtypes and states annotated by single sample subclustering on UMAP from Suppl. Fig 4A.
B. Gene expression of marker genes that affected endothelial cell clustering, revealing location-specific cell states (position shown in Suppl. Fig. 4D).
C. Expression of differential marker genes in annotated SCRINSHOT-based cell subtypes (using single sample clustering). Data from four donors.
D. Expression of the marker genes from (C) in scRNAseq data from Madissoon et al with level 3 annotations (as published). Data from eight donors. In SCRINSHOT data *TIMP1*-expressing fibroblasts are corresponding to adventitial fibroblasts by the lack of *APOE* and *TAGLN* expression, *RGCC*-expressing – to alveolar, and *FBLN1* expressing – peribronchial and/or perichondrial. *CLDN5*-high endothelial cells are corresponding to vascular capillaries g (general), *SPARCL1* – to systemic venous and arterial, as well as vascular capillary a (aerocytes) *IGFBP7* – to lymphatic endothelium, and triple positive – to pulmonary vascular endothelium. *APOE*-expressing macrophages are corresponding alveolar and *CHIT1* and dividing macrophages, and likely contain other subtypes, except interstitial, AW and CCL.

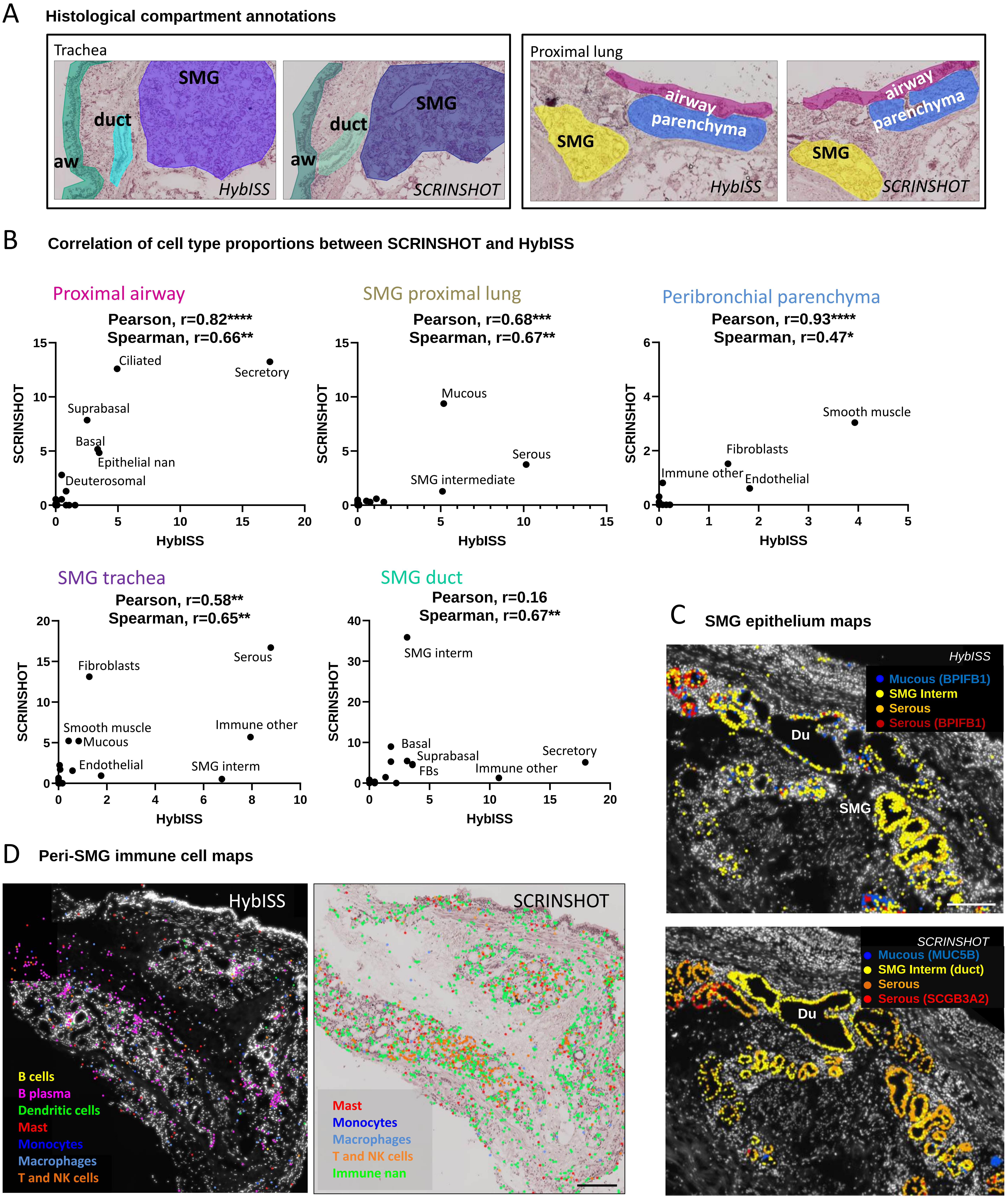

Supplementary Figure 6. Spatial validation of cell type annotation.

A. Manual annotation of histological compartments in serial sections from tracheal sample of donor 1 and proximallung sample of donor 3.

B. XY plots with correlation analysis between HybISS and Visium, SCRINSHOT and Visium, SCRINSHOT and HybISS, with percentage of detected cell types from total nuclei count per compartment. P values of corresponding correlation analyses are indicated next to r value as asterisks (* <0.05, ** <0.01, *** <0.001, ****<0.0001). Most abundant cell types of each compartment are labeled.

C. Serial sections analysed by HybISS (left) and SCRINSHOT (right) with a representative area of trachea (DAPI, white) from donor 1, containing submucosal gland cell types. SMG intermediate population in HybISS is shifted towards the smaller tubules, compared to SCRINSHOT, where SMG intermediate population is predominantly located in the duct. The SMG duct in SCRINSHOT is populated by SMG intermediate cells, whereas in HybISS the duct is composed of mixed cell types including the airway epithelium (see also Suppl. Fig. 8A, right).

D. Serial sections analysed by HybISS (left, on DAPI, white) and SCRINSHOT (right, on histological image), with a representative area of from proximal lung SMG of donor 2. Immune nan cells in SCRINSHOT are mostly colocalised with B plasma cells around the submucosal glang in HybISS, however are also presented in other regions where B plasma cells are absent. Scale bar 200 µm. Source data are provided as a Source Data file.

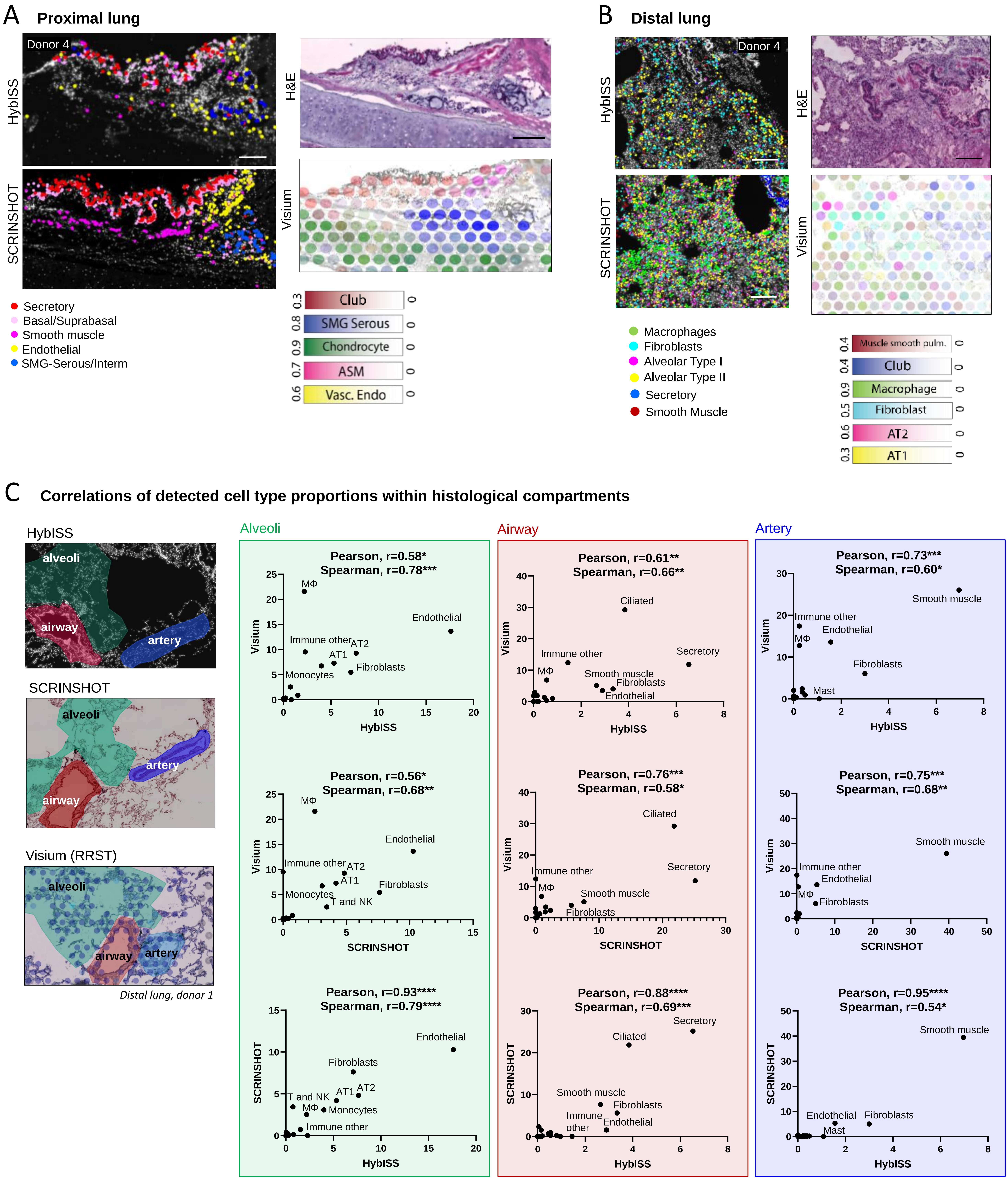

Supplementary Figure 7. Cell type map validation by gene expression using Visium, SCRINSHOT and HybISS.
A-B. Maps of the indicated cell types, detected by HybISS, SCRINSHOT and Visium in proximal (A) and distal (B) lung tissue sections. Detected cell types are shown on top of hematoxylin and eosin staining for Visium or DAPI (gray) for SCRINSHOT and HybISS, Cell types for lung Visium are detected using Stereoscope method with 80 annotated cell subtypes [1]. Scale bar 200 µm.
C. Correlations between proportions of 17 grouped cell types detected in specific tissue compartments in serial sections using three different methods. (Left) Manual annotation of histological compartments in serial sections from distal lung sample of donor 1. (Right) XY plots with correlation analysis between HybISS and Visium, SCRINSHOT and Visium, SCRINSHOT and HybISS, with percentage of detected cell types from total nuclei count per compartment. Corresponding P values from correlation analysis are indicated next to r value as asterisks (* <0.05, ** <0.01, *** <0.001, ****<0.0001). Most abundant cell types of each compartment are labeled. Source data are provided as a Source Data file.

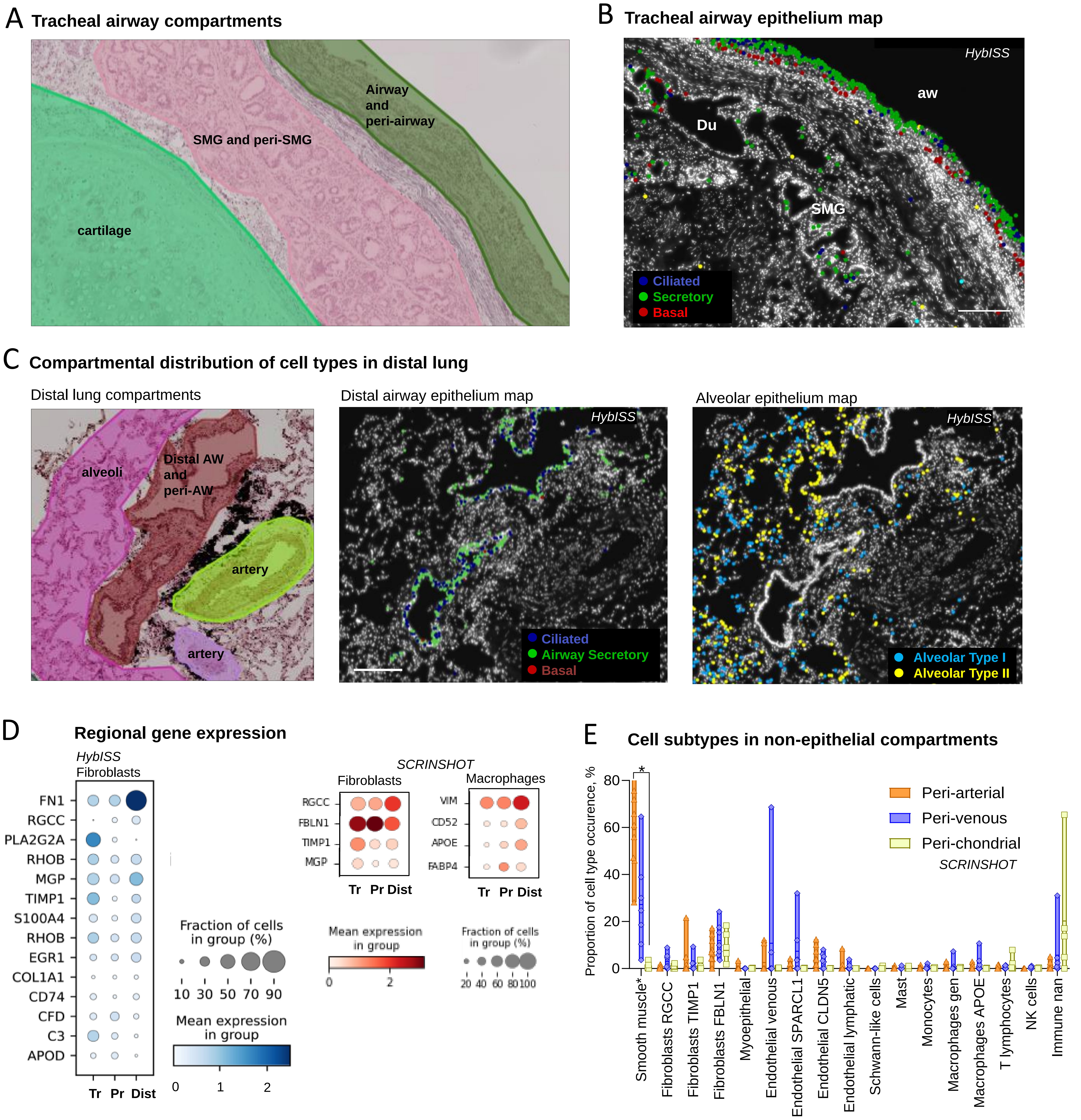

Supplementary Figure 8. Selection of histological compartments and their cell type composition.

A. Representative compartment zonation of tracheal tissue by on histological image from donor 1.

B. Airway epithelial cell type maps of trachea (DAPI, white), HybISS dataset of donor 1 trachea.

C. Representative compartment zonation of distal lung tissue on histological image (left), airway (middle) and alveolar (right) epithelial cell type type maps on the same regions (DAPI, white), HybISS dataset of donor 1 distal lung.

D. Gene expression variation by anatomical region. (Left) Dotplot showing the gene expression levels (color intensity) and the percent of positive cells for the indicated markers in the annotated fibroblasts across regions. Data from four donors, HybISS. (Right) Dotplots showing the gene expression levels (color intensity) and the percent of positive cells for the indicated markers in the annotated fibroblasts and macrophages across regions. Data from four donors, SCRINSHOT.

E. Box plot of mean cell type/subtype proportions in each of the noni-epithelial compartments from SCRINSHOT. Proportion per compartment is indicated in percent (%), combined from all regions. Bounds of box plot: minimum and maximum values; line at mean; individual points: sampled areas from three regions of four donors. Peri-arterial (n=9), peri-venous (n=7) and peri-chondrial (n=5) compartments from four donors were compared using Kruskal-Wallis test and revealed significant increase in smooth muscle cells in peri-arterial compared to peri-chondrial compartment (adjusted P=0.0013), indicated by line and asterisk (*). In all images nuclei: gray and scale bar 200 µm. Source data are provided as a Source Data file.

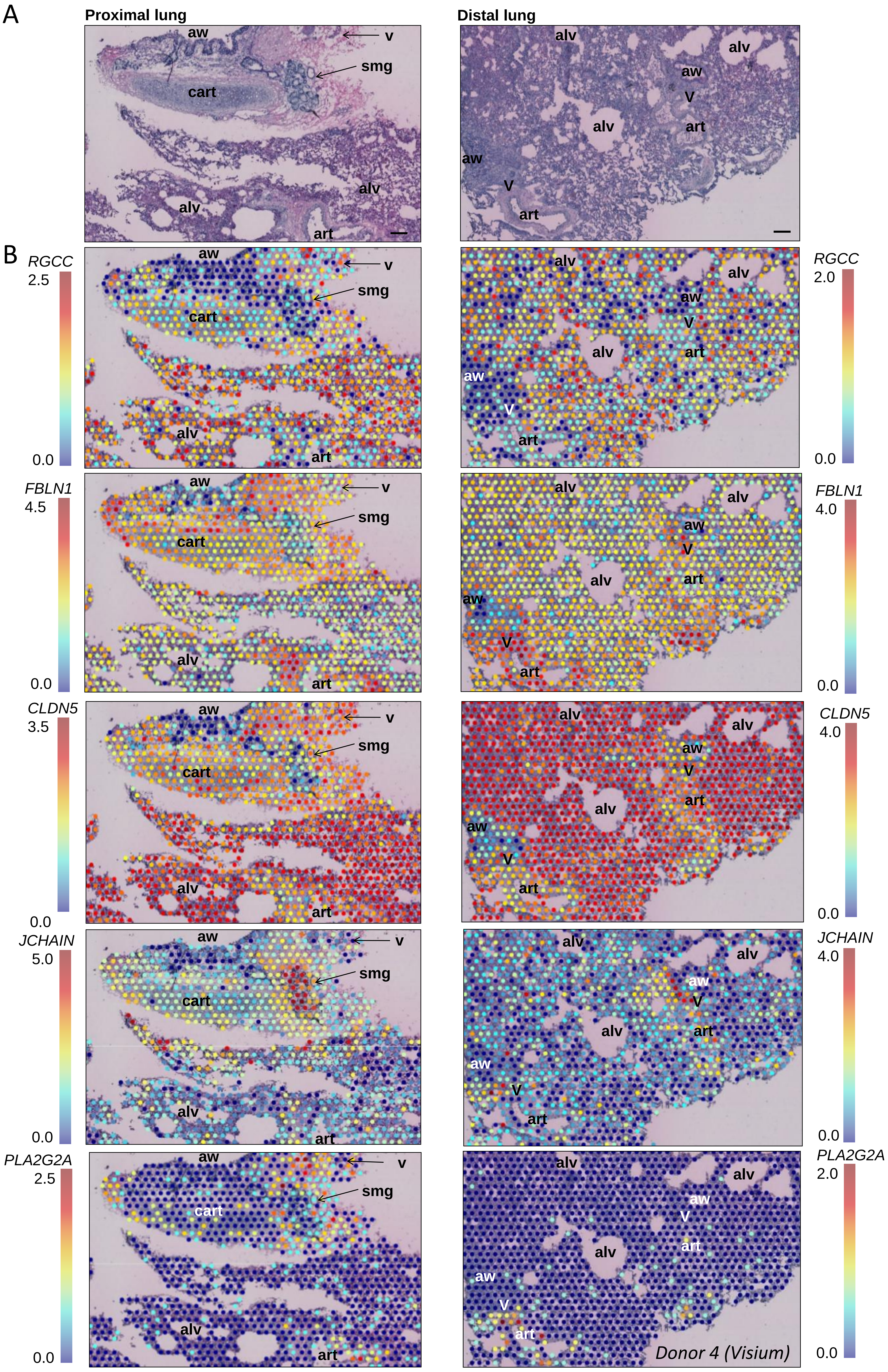

Supplementary Figure 9. Gene expression maps by RRST in alveolar and peri-epithelial neighborhoods.

A. Histological images of proximal (left) and distal (right) lung sections stained with hematoxylin and eosin from donor 4. Scale bar 200 µm.

B. Maps of gene expression levels projected on top of proximal (left) and distal (right) lungs shown in A. Data from donor 4, RRST. Arrows and letters indicate histologic compartments around submucosal gland (SMG), airways (aw), arteries (art), veins (v), cartilage (cart) and alveoli (alv). Genes expression is shown in log-normalised values obtained in Seurat. *RGCC* and *CLDN5* expression is highest in alveolar region, *FBLN1* and *PLA2G2A* – highest around blood vessels, also expressed around airways and cartilage, *JCHAIN* – around submucosal gland and distal airways.

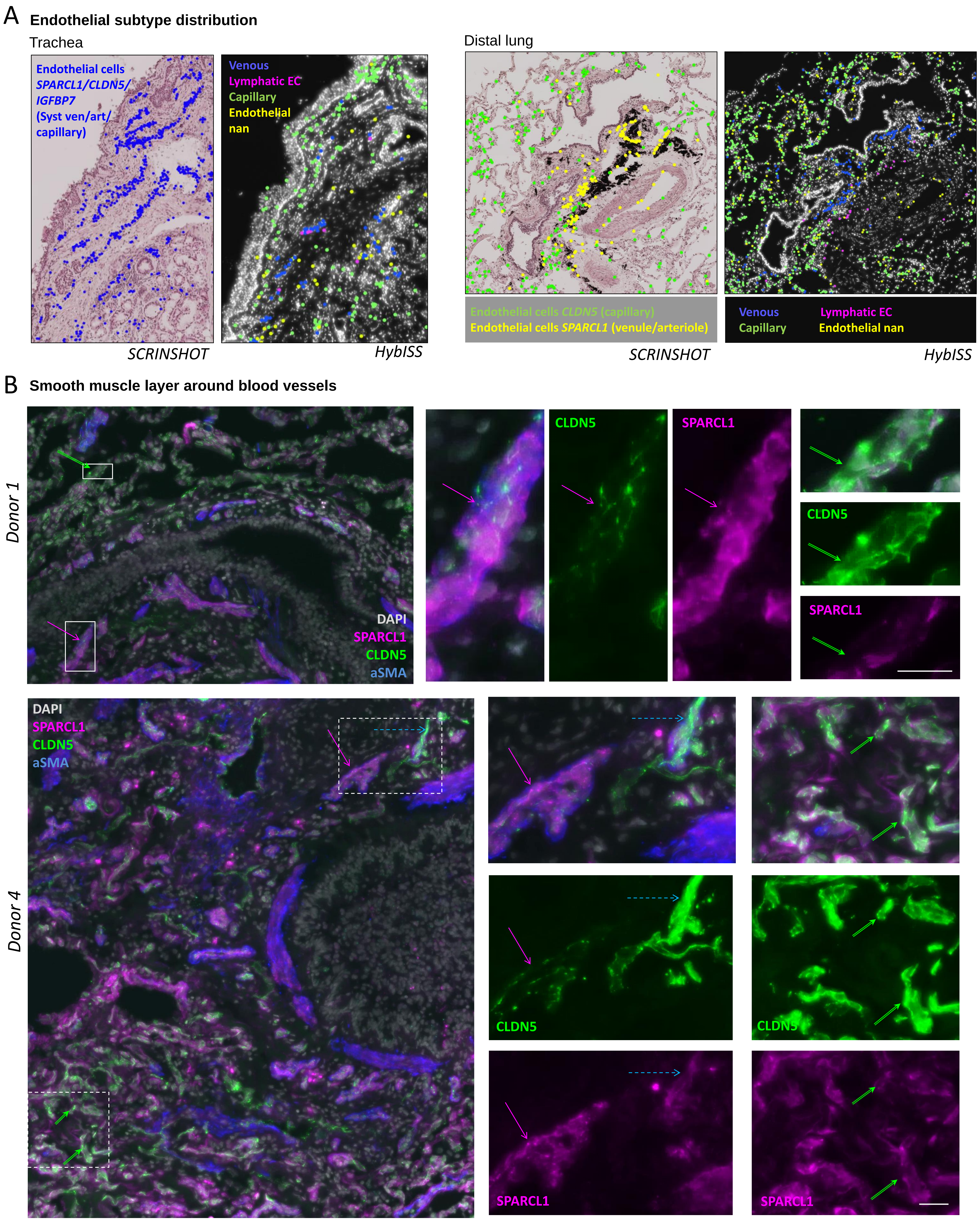

Supplementary figure 10. Endothelial cell subtype characterisation.

A. Serial sections analysed by SCRINSHOT (left, on histological image) and HybISS (right, on DAPI, white) with a representative area of from (left) trachea of donor 4, and (right) distal lung of donor 1, containing endothelial cell types. Triple-positive endothelial cell cluster in SCRINSHOT (*SPARCL1/CLDN5/IGFBP7*) is colocalised with mostly venous and capillary cells in HybISS, and annotated pulmonary venous/arterial/capillary by their gene expression. *CLDN5*-positive cluster in SCRINSHOT is colocalised with mostly capillary cells in HybISS in alveolar region, and is therefore annotated capillary, whereas *SPARCL1*-positive cluster in SCRINSHOT is colocalised with mostly venous cells in HybISS in peribronchial region, and is annotated systemic venous/arterial.

B. Immunofluorescent staining of endothelium and smooth muscle using SPARCL1 (magenta), CLDN5 (green) and alpha smooth muscle actin (aSMA, blue) antibodies in the distal lung of donor 1 (top) and donor 4 (bottom). Squared areas are zoomed in on the right side. Arrows indicate the SPARCL1-high vessels (plain arrows) and double-positive vessels (dashed arrow) surrounded by thin layer of aSMA near the airways, and CLDN5-high vessels (double arrows) in the alveoli. Scale bar 20 µm.

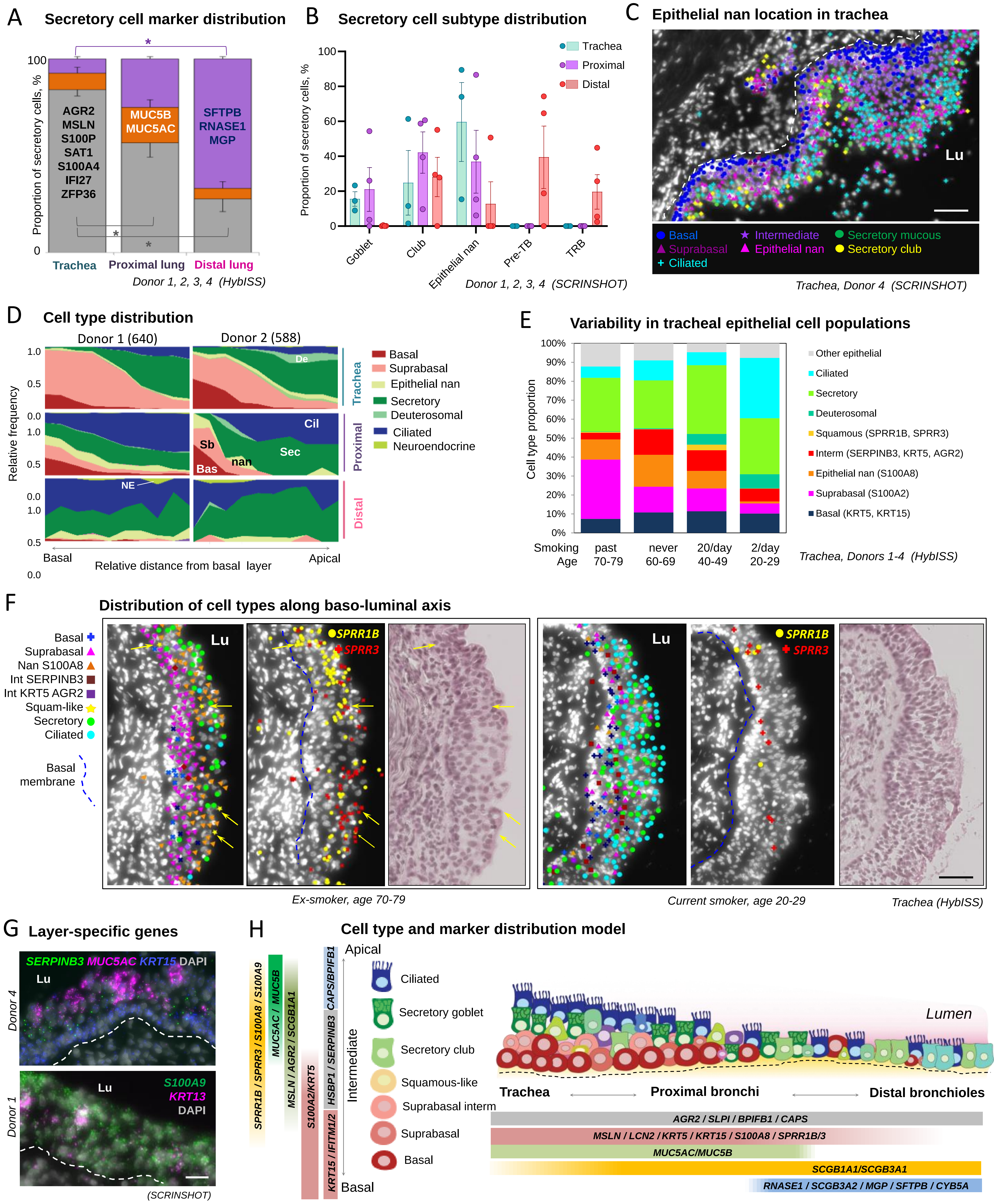

Supplementary Figure 11. Defining epithelial cell states by gene expression and spatial distribution.

A. Stacked bar plot of quantification of grouped subtypes of secretory cells in the bronchial epithelium from four donors (n=4), detected by HybISS. Error bars: standard deviation. Significant differences according to two-way ANOVA followed by Tukey's multiple comparisons test, are highlighted with asterisk, color-coded according to bars, adjusted P values are as follows: for *SFTPB/MGP/RNASE* expressing cells in trachea vs distal lung P=0.0178, for *AGR2* expressing cells in trachea vs proximal P=0.0207, trachea vs distal P=0.0174, n=4 (data from four biological replicates).

B. Bar plot of quantification of goblet, club, pre-terminal bronchiole (pre-TB), terminal bronchiole (TRB) and other secretory cells in the epithelium of four donors (n=3 for trachea, n=4 for lung), detected by SCRINSHOT. Error bars: standard error.

C. Representative map of epithelial cell types in tracheal epithelium of donor 4, based on SCRINSHOT. Scale bar 50 µm.

D. Area plots representing the relative apical-basal cell type distribution across regions according to HybISS (donor 1 and 2). X axis: relative distance of cells from the basal membrane, in addition to Fig. 3D. Y axis: relative frequency of cell types.

E. Stacked bar plot showing the percent of basal and suprabasal cell states in tracheal samples in four analysed donors, indicating the high variability in the abundance of suprabasal, squamous-like, and intermediate cell states.

(continiued on the next page)

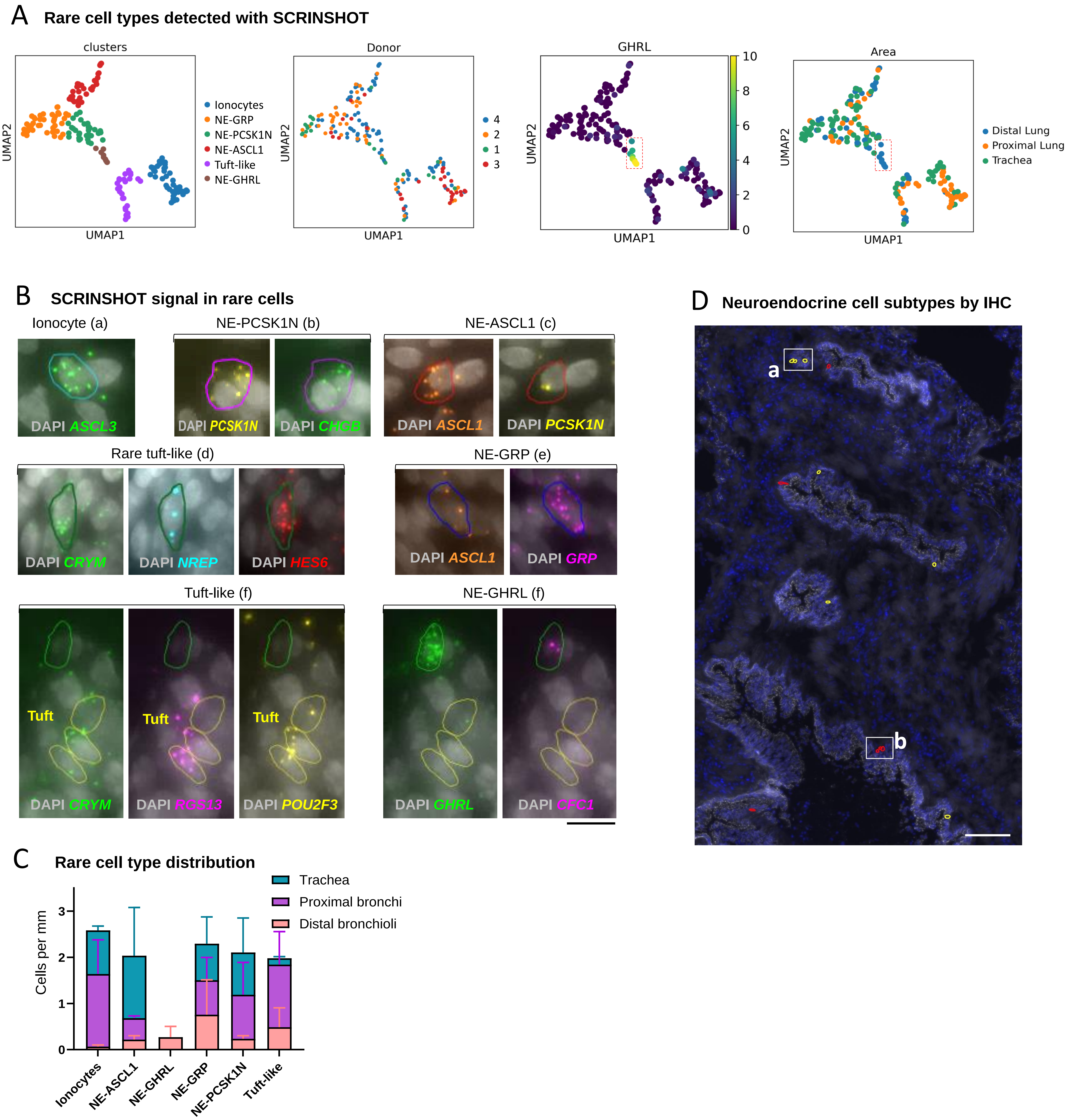

Supplementary Figure 12. Rare epithelial cell diversity and location.

A. UMAP plots of clustered rare cells annotated by positivity to markers. GHRL gene expression is shown in cells from distal lung in at least three donors.

B. SCRINSHOT signal of rare cell type marker genes plotted on top of nuclei (DAPI, grey) in the indicated regions in Figure 4 (A). The images show both high (>10 dots per cell for *ASCL3*, *GHRL* and *GRP*) and low (1-2 dots per cell) for *CFC1*, *POU2F3*. Variable levels are shown for *ASCL1* and *PCSK1N*. Scale bar 10 µm.

C. Stacked bar plot of the average number of the detected rare cells per 1 mm length of basal membrane from four donors. Bar: mean; error bars: standard error from 2 biological replicates. NE-*GHRL*^{pos} cells appearing only in distal lung.

D. Immunofluorescent staining of distal lung sample of donor 4 for CDH1 (white) on nuclei (DAPI, blue), indicating manually labeled neuroendocrine cells positive for GHRL (yellow) and GRP (red). Zoomed squared regions in Fig. 4C. Scale bar 100 µm. Source data are provided as a Source Data file.

Supplementary Figure 11. Defining epithelial cell states by gene expression and spatial distribution (continued from previous page).

F. Representative images of tracheal epithelium from two donors, showing (left) the maps of the indicated cell types, (middle) the *SPRR1B* and *SPRR3* squamous marker detected transcripts on top of nuclei (DAPI, white) and (right) hematoxylin and eosin staining after HybISS. Arrows: squamous-like cells. Scale bar 50 µm. Lu – lumen, dashed line indicates approximate location of basal membrane.

G. Images of SCRINSHOT signal of representative transcripts defining cell states in tracheal epithelium on top of nuclei (DAPI, white). (top) *KRT15*: basal layer and *SERPINB3*: suprabasal layer, *MUC5AC*: luminal layer. (bottom) *KRT13* intermediate layer and *S100A9*: all layers. Lu – lumen, dashed line: approximate location of basal membrane. Scale bar 20 µm.

H. Schematic summary of cell type distribution and gene expression patterns along proximo-distal and apical-basal axes. Source data are provided as a Source Data file.

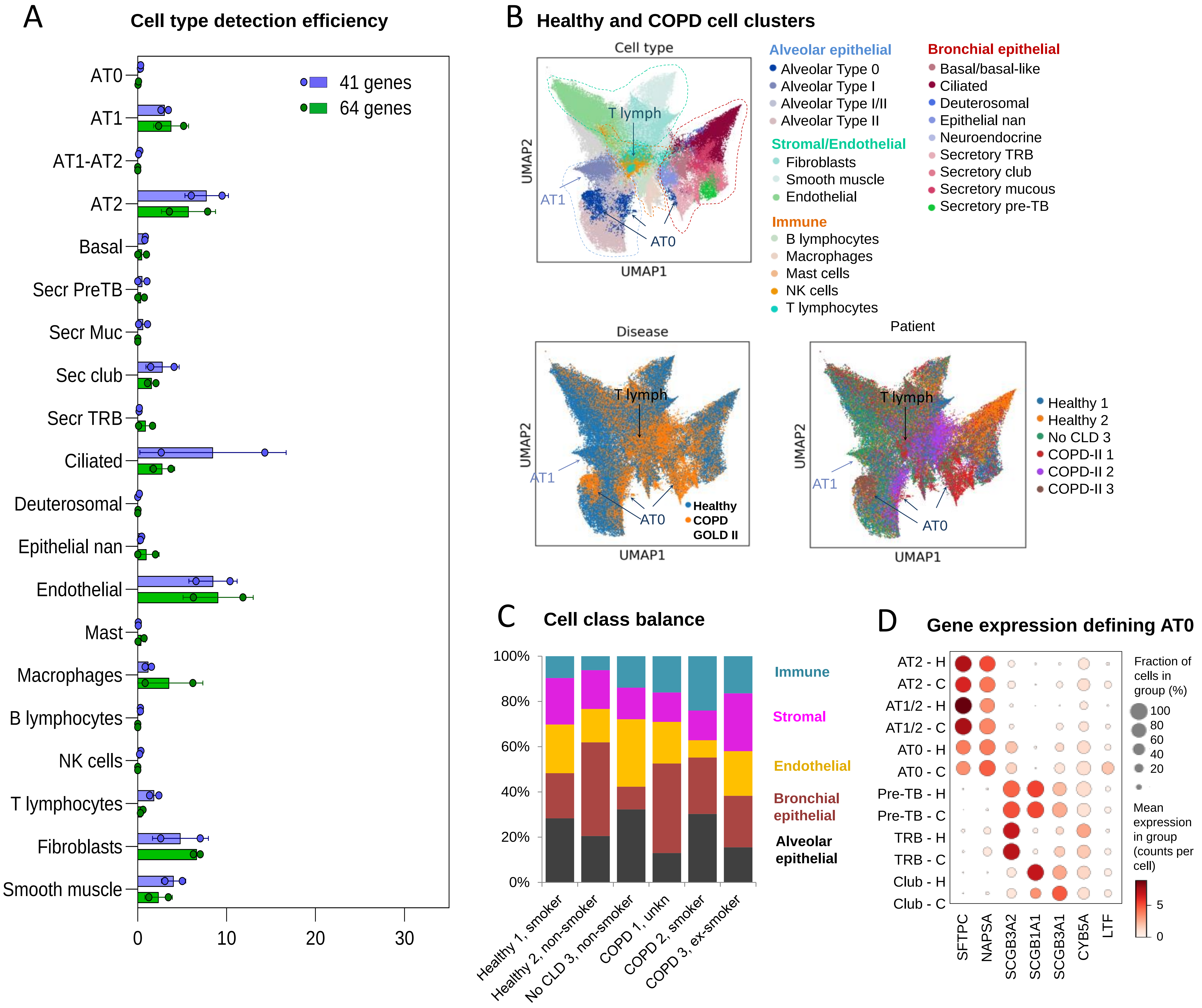

Supplementary Figure 13 Quantification of COPD-related changes in cell types and states.

A. Bar plot of the cell type proportions among total cell counts (including negative cells) from donors 2 and 4 from atlas (defined by 64 genes) in comparison with the COPD experiment (defined by 41 genes, with extra markers for epithelial cells), n=2. Bars: mean values; error bars: standard deviation; individual points: biological replicates.

B. UMAP plots of cell type clusters (left), health status (middle) and donors/patient identities (right), from distal lung biopsies analyzed by SCRINSHOT.

C. Stacked barplot of the proportions of the indicated cell classes in the analyzed donors.

D. Dotplot showing the mean expression of the indicated genes in the AT2-related clusters compared to distal secretory cell types in healthy (-H) and COPD (-C) subsets. Color intensity: number of counts per cell. Dot size: percentage of positive cells. Source data are provided as a Source Data file.

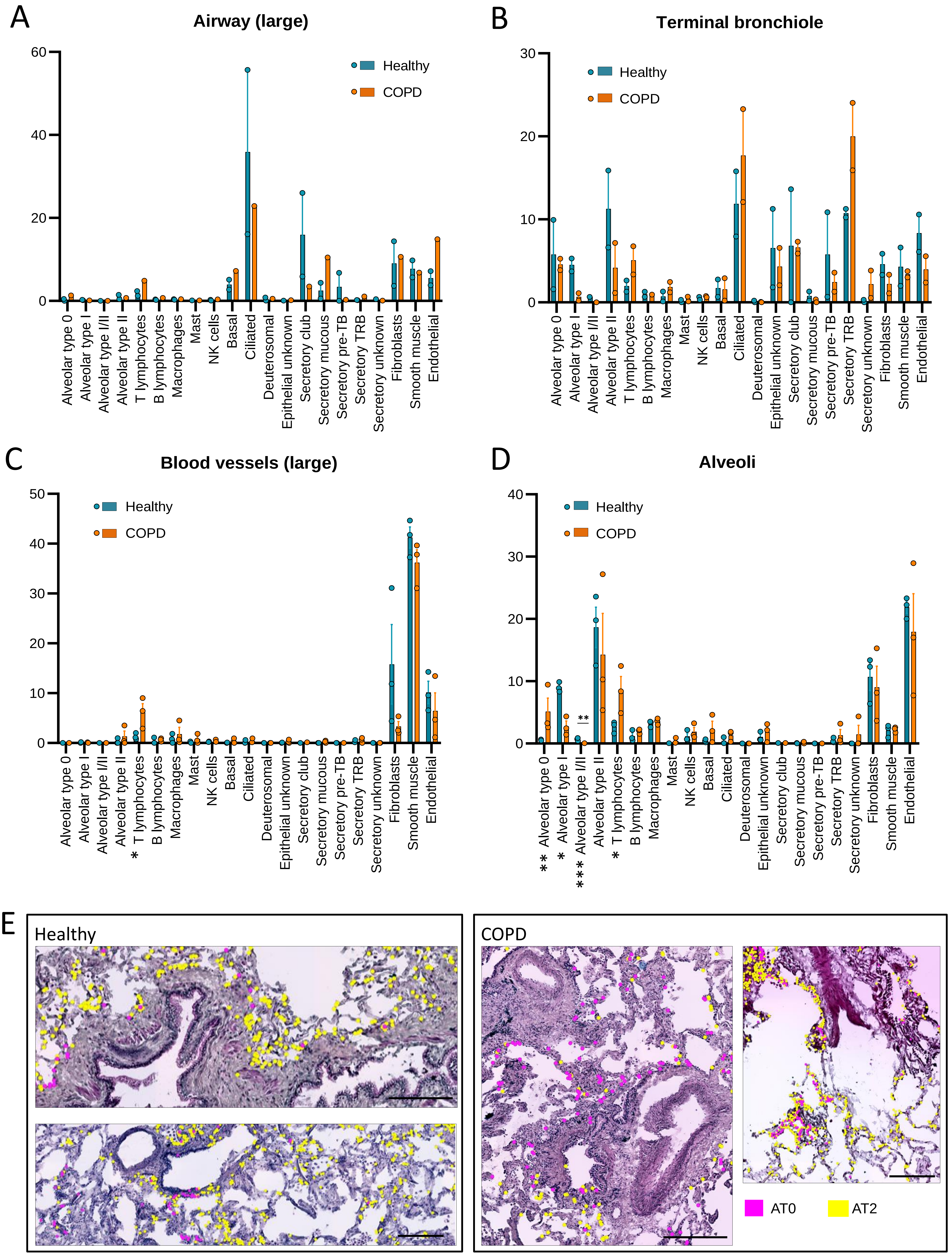

Supplementary Figure 14. Compartment-specific cell type distribution in healthy and COPD lungs.
A-D. Bar plots with individual data points indicating the proportions of each cell type within a particular tissue compartment in healthy/no CLD and COPD samples. Bars: mean values; error bars: SEM, individual points: biological replicates. Significant differences observed according to multiple t-test (17) of logit-transformed data with Holm-Sidak correction, n=3, are indicated with a bar with asterisks. For alveolar compartment adjusted P value for ATI/II is P=0.0113. Non-adjusted P values are: AT0=0.0085, ATI: 0.0161, T lymphocytes: 0.033. The significant differences after a direct cell type comparison without the correction between healthy and COPD samples are labeled by asterisks at the cell type name.
E. Maps of AT0 (magenta) and AT2 (yellow) cells on top of histological images in healthy and COPD samples. Scale bar 200 µm. Source data are provided as a Source Data file.

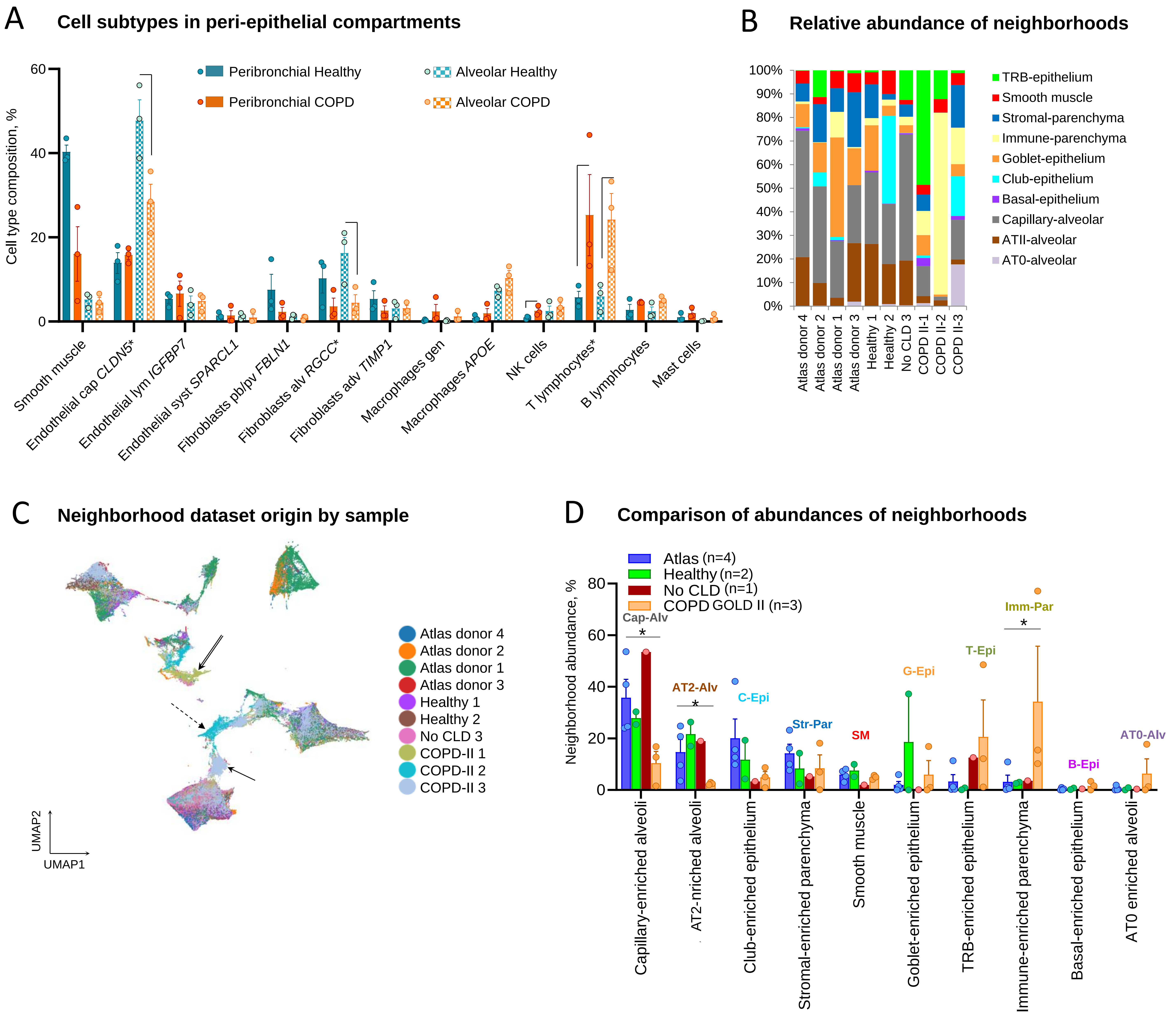

Supplementary Figure 15. Cell type and subtype distribution in healthy and COPD lung.

A. Bar plot of the mean proportions (%) of the indicated cell type populations in the annotated peri-epithelial compartments. Bars: mean values; error bars: SEM, individual points: biological replicates. No significant differences observed, according to multiple t-test (17) of logit-transformed data with Holm-Sidak correction, n=3. The direct cell type comparison without the correction revealed significant differences between healthy and COPD samples in the following cell types: endothelial CLDN5 (P=0.0445), fibroblasts RGCC (P=0.046) in alveolar regions, NK cells in peribronchial regions (P=0.0449), and T lymphocytes (P=0.0424 and P=0.0436) in both regions (labeled by asterisks at the cell type name).
B. Stacked barplot of the proportions of detected cellular neighborhood clusters (Figure 5C) by donor/patient.
C. UMAP plot of the detected cellular neighborhood clusters (Figure 5C) colored by donor/patient. Metadata presented in supplementary table 1.
D. Bar plot showing the relative abundance mean values of the indicated cell neighborhoods across all datasets (Atlas, COPD, healthy and no CLD). Values on Y axis indicate the percentage of cell numbers contributing to each neighborhood among all other neighborhood-annotated cells for each sample. Bars: mean values; error bars: SEM, individual points: biological replicates. Significant differences are highlighted with asterisk (*, p≤0.05), according to one-way ANOVA of logit-transformed data (0 values changed to the half of minimal detection limit values) followed by Dunnet's posthoc with Atlas group used as a control, and Healthy/No CLD treated as one group. Adjusted P values are as follows: AT2-Alv Atlas vs COPD: 0.0265, Cap-Alv Atlas vs COPD: 0.0499, Imm-Par Atlas vs COPD: 0.0297. TRB – terminal respiratory bronchiole. Source data are provided as a Source Data file.

Region	Donor 1	Donor 2	Donor 3	Donor 4
Trachea (1)	SCRINSHOT (Cell Type, Rare), HybISS	SCRINSHOT (Cell Type, Rare-quantified), HybISS	HybISS	SCRINSHOT (Cell Type, Rare-quantified), HybISS
Proximal lung (2)	SCRINSHOT (Cell Type, Rare), HybISS	SCRINSHOT (Cell Type), HybISS	SCRINSHOT (Cell Type, Rare-quantified), HybISS	SCRINSHOT (Cell Type, Rare-quantified), HybISS, VISIUM 3D, RRST
Distal lung (3a)	not used	HybISS	HybISS	not used
Distal lung (3b)	SCRINSHOT (Cell Type, Rare), HybISS, VISIUM, RRST	not used	HybISS, SCRINSHOT (Cell Type, Rare)	SCRINSHOT (Cell Type, Rare-quantified), HybISS
Distal lung (3c)	not used	SCRINSHOT (Cell Type, Rare-quantified, COPD), HybISS	not used	RRST, SCRINSHOT (COPD)

Supplementary Table 1. Spatial transcriptomics methods used on each sample.

Cell type	Criteria
Ionocytes	ASCL3 (>2 dots) and positivity for either SEC11C or ATP6V0B
T cells	CD3D and CD8A double positivity
NK cells	Two of these genes (CD3E, CCL5, GNLY, NKG7) detected and either CD3D or CD8A expressed
Tuft cells	RGS13 (>2 dots) and positivity for BMX
Aerocytes	Positivity for CLDN5 and/or RAMP2 within EDNRB+ cluster of mesenchymal cells
Squamous-like SPRR3	SPRR3 (>3 dots) within Epithelial nan (S100A4) cluster
Squamous-like SPRR1B	SPRR1B (>3 dots) within Suprabasal intermediate KRT5-AGR2 cluster
Rare tuft-like cells	NREP (>2 dots) and HES6 (>2 dots)

Supplementary Table 2. Criteria for selecting additional cell types in HybISS.
