# Supplementary material for: Spatial single-cell atlas reveals regional variations in healthy and diseased human lung
Source: Nat Commun. 2025 Nov 5;16:9745. doi: 10.1038/s41467-025-65704-0 (PMC12589588; doi:10.1038/s41467-025-65704-0)
Supplement: Supplementary file 2 — Description of Additional Supplementary Files [file 41467_2025_65704_MOESM2_ESM.pdf]

Description of Additional Supplementary Files

Supplementary Data 1. Donor details.

Metadata for organ donors of healthy lungs and cancer patients with lobectomies regarding their health status, reason for biopsy, gender, age group, smoking status, ethnicity, and cite of biopsy. Information also includes anonymized atlas donor numbers, COPD sample ID and biopsy cite region code.

Supplementary Data 2. HybISS probe panel.

List of marker genes selected either using Spapros or from previously published information. PubMed ID (PMID) stated in column C. Information includes gene name, characteristic cell type and probe details from Cartana, such as probe name, ID (LbarID), barcode and the number of padlock probes (PLP) used. Padlock probe sequences can be obtained or ordered from 10x Genomics.

Supplementary Data 3. HybISS cell annotation.

Annotated cell class, type and subtype hierarchy, and criteria for cluster annotations by their differentially expressed genes. The list of cell types includes manual annotations by positivity, which is described in detail in Supplementary Table 2.

Supplementary Data 4. SCRINSHOT cell type markers.

List of marker genes selected either using Spapros or from previously published information (PMID stated in column G) including gene name, cell type of expression. Padlock probe sequences can be viewed in Supplementary Data Excel 7.

Supplementary Data 5. SCRINSHOT cell annotation.

Annotated cell class, type and subtype hierarchy, and criteria for cluster annotations by their differentially expressed genes.

Supplementary Data 6. HybISS-to-scRNAseq cell type dictionary.

The dictionary used to combine and modify cell type annotations for integration mapping shown in Supplementary Figure 3A.

Supplementary Data 7. SCRINSHOT padlock probe sequences.

List of padlock probes used for SCRINSHOT, including the name of detected gene, recognized mRNA sequences, padlock probe sequences and their melting temperatures, and barcode sequences used for detection probes.

Supplementary Data 8. SCRINSHOT marker probe panel.

List of marker genes used in three independent SCRINSHOT experiments: general cell type detection (Cell Type panel), rare epithelial cell detection (Rare cell panel) and distal lung cell type detection used for COPD and control samples (COPD panel).
