# Supplementary material for: Spatial single-cell atlas reveals regional variations in healthy and diseased human lung
Source: Nat Commun. 2025 Nov 5;16:9745. doi: 10.1038/s41467-025-65704-0 (PMC12589588; doi:10.1038/s41467-025-65704-0)
Supplement: Supplementary file 4 — Reporting Summary [file 41467_2025_65704_MOESM4_ESM.pdf]

nature portfolio

Corresponding author(s): Christos Samakovlis

Last updated by author(s): Sep 19, 2025

Reporting Summary

Nature Portfolio wishes to improve the reproducibility of the work that we publish. This form provides structure for consistency and transparency in reporting. For further information on Nature Portfolio policies, see our Editorial Policies and the Editorial Policy Checklist.

Statistics

For all statistical analyses, confirm that the following items are present in the figure legend, table legend, main text, or Methods section.

n/a	Confirmed	
☐	☒	The exact sample size (*n*) for each experimental group/condition, given as a discrete number and unit of measurement
☒	☐	A statement on whether measurements were taken from distinct samples or whether the same sample was measured repeatedly
☐	☒	The statistical test(s) used AND whether they are one- or two-sided<br>*Only common tests should be described solely by name; describe more complex techniques in the Methods section.*
☐	☒	A description of all covariates tested
☐	☒	A description of any assumptions or corrections, such as tests of normality and adjustment for multiple comparisons
☐	☒	A full description of the statistical parameters including central tendency (e.g. means) or other basic estimates (e.g. regression coefficient) AND variation (e.g. standard deviation) or associated estimates of uncertainty (e.g. confidence intervals)
☐	☒	For null hypothesis testing, the test statistic (e.g. *F*, *t*, *r*) with confidence intervals, effect sizes, degrees of freedom and *P* value noted<br>*Give P values as exact values whenever suitable.*
☒	☐	For Bayesian analysis, information on the choice of priors and Markov chain Monte Carlo settings
☐	☒	For hierarchical and complex designs, identification of the appropriate level for tests and full reporting of outcomes
☐	☒	Estimates of effect sizes (e.g. Cohen's *d*, Pearson's *r*), indicating how they were calculated

Our web collection on statistics for biologists contains articles on many of the points above.

Software and code

Policy information about availability of computer code

Data collection	HybISS data was collected with a Zeiss Axio Imager Z2 (Carl Zeiss Microscopy, GmbH) equipped with Zeiss Plan-Apochromat 20x/0.8 objective, multi-slide stage (PLine) and a Lumencor spectra X light engine LED source, quad band chroma 89402 and 89403, and single band Zeiss 38HE. Images were obtained with ORCA-Flash4.0 LT Plus sCMOS camera (Hamamatsu Photonics K.K.) using Zen Blue 2.5 software (Carl Zeiss Microscopy, GmbH).<br>SCRINSHOT and immunofluorescence data were collected using Zeiss Axio Observer Z2 with a Colibri 7 LED light source (Carl Zeiss Microscopy, GmbH, 423052-9770-000) equipped with a Zeiss 20x/0.75 Plan-Apochromat and a Zeiss AxioCam 506 Mono digital camera, and Chroma filters: 49000, 49003, 49304, 49307, 49310, and 49007. Images were acquired and processed using Zen Blue 2.5 software (Carl Zeiss Microscopy, GmbH).<br>RNA quality evaluation was performed using the RNeasy Mini kit (Qiagen, Catalog number 74104). Extracted total RNA was measured using the Agilent Bioanalyzer (Agilent, RNA 6000 Pico kit, Part number 5067–1513) to obtain RINs.<br>Visium and RRST data were collected following 10× Genomic Visium Spatial Gene Expression protocol (User Guide, CG000239 Rev F, Product number 1000187). Libraries were sequenced on Next-seq2000 (Illumina). Data was generated from raw sequencing files using the 10x Genomics Space Ranger software v1.2.1 or 1.3.1 and R v.4.1.3.
Data analysis	For Visium sequenced libraries were processed using Space Ranger software (version 1.2.1 for standard V isium data and version 1.3.1 for RRST data, 10× Genomics). Analysis was carried out using R. Custom code and data is available at https://github.com/ludvigla/DiscovAir_data_explorer/ (v1.0) and https://github.com/ludvigla/RRST (v1.0.0)<br>For HybISS data analysis and processing pipelines can be accessed here: https://github.com/Moldia/adult_lung_DiscovAIR_spatial, accession code 10.5281/zenodo.17175597. Python package called starfish (https://spacetxstarfish.readthedocs.io/en/latest/) and the decoding code can be found at https://github.com/Moldia/ISS_decoding (commit feeade7).<br>For SCRINSHOT probe design RStudio 4.0.3. was used with this code: https://github.com/alexandra-firsova/Barcode-design-of-padlock-

probes/, accession code: 10.5281/zenodo.17171960, as well as previously described pipelines: https://github.com/AlexSount/SCRINSHOT_scripts_for_EMBO_course/blob/main/padlock_probe_design_v6_empty_stable_backbone.R (commit 9d02655) and https://github.com/HelmholtzAI-Consultants-Munich/oligo-designer-toolsuite (v0.1.3). For image analysis ImageJ Fiji 1.53c, and CellProfiler 3.1.9. were used. SCRINSHOT analysis code can be obtained here: https://github.com/alexandra-firsova/Cell-analysis-in-SCRINSHOT, DOI 10.5281/zenodo.17167875.

For manuscripts utilizing custom algorithms or software that are central to the research but not yet described in published literature, software must be made available to editors and reviewers. We strongly encourage code deposition in a community repository (e.g. GitHub). See the Nature Portfolio guidelines for submitting code & software for further information.

Data

Policy information about availability of data

All manuscripts must include a data availability statement. This statement should provide the following information, where applicable:

- Accession codes, unique identifiers, or web links for publicly available datasets
- A description of any restrictions on data availability
- For clinical datasets or third party data, please ensure that the statement adheres to our policy

The HybISS data and analysis generated in this study have been deposited in the github database under accession code DOI: 10.5281/zenodo.17175597. The raw SCRINSHOT data images were deposited in BioImage Archive https://www.ebi.ac.uk/biostudies/bioimages/studies/ under accession codes S-BIAD2307 for healthy lung atlas, S-BIAD2308 for rare cells and S-BIAD2310 for COPD cohort. All processed data (SCRINSHOT and HybISS) and cell type map files, gene expression matrices and coordinates, immunofluorescent and histological images are available by the following link https://www.ebi.ac.uk/biostudies/studies/S-BSST2188 with accession number 10.6019/S-BSST2188. The statistical data generated in this study are provided in the Supplementary Source Data Excel files and at https://www.ebi.ac.uk/biostudies/studies/S-BSST2189. The interactive viewers of cell types and links to all data are available under the following link: https://github.com/alexandra-firsova/Human-lung-cell-atlas (DOI: 10.5281/zenodo.17179727). Source data are provided with this paper.

Research involving human participants, their data, or biological material

Policy information about studies with human participants or human data. See also policy information about sex, gender (identity/presentation), and sexual orientation and race, ethnicity and racism.

Reporting on sex and gender	Sex of participants is specified in the provided supplementary information tables. Gender information was not available.
Reporting on race, ethnicity, or other socially relevant groupings	Race and ethnicity of participants is provided where available in the supplementary information tables.
Population characteristics	Participants age, known health condition and smoking status is provided in the supplementary information tables.
Recruitment	Participants were recruited based on the availability to our research groups in the sample collection location, based in the UK and Germany.
Ethics oversight	For healthy donors informed consent from the families and approval from NRES Committee of East of England, Cambridge South, was obtained (15/EE/0152). For the diseased sample analysis, tissues were collected with written informed consent from all patients and with ethics approval from the ethics committee of the Ludwig Maximilian University of Munich (#330-10, #19-629 and #19-630).

Note that full information on the approval of the study protocol must also be provided in the manuscript.

Field-specific reporting

Please select the one below that is the best fit for your research. If you are not sure, read the appropriate sections before making your selection.

☒ Life sciences ☐ Behavioural & social sciences ☐ Ecological, evolutionary & environmental sciences

For a reference copy of the document with all sections, see nature.com/documents/nr-reporting-summary-flat.pdf

Life sciences study design

All studies must disclose on these points even when the disclosure is negative.

Sample size	No statistical method was used to predetermine sample size. Sample sizes were suggested in previous publications (https://doi.org/10.1038/s41588-022-01243-4 and https://doi.org/10.1183/13993003.02057-2021).
Data exclusions	For healthy cohort samples with signs of severe inflammation or pathology were excluded based on histological analysis. For mRNA analysis samples with low quality of mRNA (based on signal strength analysed with RIN and SCRINSHOT) were excluded.
Replication	Four biological replicates were used for healthy cohort, and three biological replicates were used for COPD cohort. From the initial cohort of six control donors, we excluded two after identifying previously unreported pathological alterations. This rigorous quality control ensured that the remaining four samples truly represented pathology-free tissue. Although these four donors differed in age, sex, and smoking history, our analyses focused on features that were consistently observed across all of them. Therefore, we concluded that increasing the number of

samples would not substantially alter the main conclusions, as the reported findings are those robust to inter-individual variability. While the sample size does not capture the full heterogeneity of stage II COPD, the primary aim of our study was to investigate COPD-associated changes at the single-cell and spatial neighborhood levels — an aspect not previously described.

Randomization	Samples were allocated to experimental groups based on special assessment from medical professionals and according to their diagnosis.
Blinding	Blinding was achieved via combination of datasets and performing statistical analysis of the whole data cohort. For data collection, samples were given random numbers.

Reporting for specific materials, systems and methods

We require information from authors about some types of materials, experimental systems and methods used in many studies. Here, indicate whether each material, system or method listed is relevant to your study. If you are not sure if a list item applies to your research, read the appropriate section before selecting a response.

Materials & experimental systems

n/a	Involved in the study
☐	☒ Antibodies
☒	☐ Eukaryotic cell lines
☒	☐ Palaeontology and archaeology
☒	☐ Animals and other organisms
☒	☐ Clinical data
☒	☐ Dual use research of concern
☒	☐ Plants

Methods

n/a	Involved in the study
☒	☐ ChIP-seq
☒	☐ Flow cytometry
☒	☐ MRI-based neuroimaging

Antibodies

Antibodies used	anti-GHRL rat monoclonal antibody (R&D Systems, MAB8200-SP, clone: 883622, wd:1.25 ug/ml, Lot: CILU0220021)<br>anti-GRP rabbit polyclonal antibody (Bioss, bs-0011R, wd:1:200, Lot: AI08112480)<br>anti-E-Cadherin mouse Alexa Fluor 555 (BD Biosciences, 560064, clone: 36/E-Cadherin, wd: 1:100, Lot: 4337645).<br>anti-alpha Smooth Muscle Actin (ACTA2) eFluor660 mouse monoclonal antibody (Thermo, 50-9760-82, clone 1A4, wd:1:100, Lot: 4347892)<br>anti-SPARCL1 goat polyclonal antibody, (BioTechne, R&D, A2728, wd: 2 ug/ml, Lot: VHT0323061)<br>anti-CLDN5 Alexa Fluor 488 mouse monoclonal antibody, (Thermo, 352588, clone 4C3C2, wd: 1:100, Lot: UF285712)
Validation	anti-GHRL: manifacturer provides information and citations regarding the species reactivity and usage on tissue sections for immunohistochemistry/immunofluorescence. https://www.rndsystems.com/products/human-mouse-ghrelin-antibody-883622_mab8200<br>anti-GRP: manifacturer provides information and citations regarding the species reactivity and usage on tissue sections for immunohistochemistry/immunofluorescence. https://www.biossusa.com/products/bs-0011r<br>anti-E-Cadherin: manifacturer provides information and citations regarding the species reactivity and usage on tissue sections for immunohistochemistry/immunofluorescence. https://www.bdbiosciences.com/en-se/products/reagents/microscopy-imaging-reagents/immunofluorescence-reagents/alexa-fluor-555-mouse-anti-e-cadherin.560064<br>anti-alpha Smooth Muscle Actin: manifacturer provides information and citations regarding the species reactivity and usage on tissue sections for immunohistochemistry/immunofluorescence. https://www.thermofisher.com/antibody/product/Alpha-Smooth-Muscle-Actin-Antibody-clone-1A4-Monoclonal/50-9760-82<br>anti-SPARCL1: manifacturer provides information and citations regarding the species reactivity and usage on tissue sections for immunohistochemistry/immunofluorescence. https://www.rndsystems.com/products/human-sparc-like-1-sparcl1-antibody_af2728<br>anti-CLDN5: manifacturer provides information and citations regarding the species reactivity and usage on tissue sections for immunohistochemistry/immunofluorescence. https://www.thermofisher.com/antibody/product/Claudin-5-Antibody-clone-4C3C2-Monoclonal/352588

Plants

Seed stocks	NA
Novel plant genotypes	*Describe the methods by which all novel plant genotypes were produced. This includes those generated by transgenic approaches, gene editing, chemical/radiation-based mutagenesis and hybridization. For transgenic lines, describe the transformation method, the number of independent lines analyzed and the generation upon which experiments were performed. For gene-edited lines, describe the editor used, the endogenous sequence targeted for editing, the targeting guide RNA sequence (if applicable) and how the editor was applied.*
Authentication	*Describe any authentication procedures for each seed stock used or novel genotype generated. Describe any experiments used to assess the effect of a mutation and, where applicable, how potential secondary effects (e.g. second site T-DNA insertions, mosiacism, off-target gene editing) were examined.*
